# Supplementary material for: Growth Regulation in the Larvae of the Lepidopteran Pieris brassicae: A Field Study
Source: Insects. 2023 Feb 9;14(2):167. doi: 10.3390/insects14020167 (PMC9965483; doi:10.3390/insects14020167)
Supplement: Supplementary file 1 [file insects-14-00167-s001.zip › Baraldi et al Supplementary/Baraldi et al Supplementary Table S1.pdf]

Table S1. Meteorological data for the period of rearing of the larvae (L1-L5) of *Pieris brassicae*. Development events and collection activities are shown. egg transfer: placement of the egg clutch on a leaf of the associated cabbage plant.

event/action	date	temperature °C			relative humidity %	
		min	max	mean	min	max
deposition	03/06/21	11.8	29.1	20.1	43	100
egg transfer	04/06/21	14.7	29.0	22.1	47	100
	05/06/21	15.3	29.9	23.1	41	99
	06/06/21	16.5	26.1	20.5	56	100
hatching	07/06/21	17.6	28.9	21.7	50	100
L1 collection	08/06/21	17.0	31.4	23.9	36	98
	09/06/21	18.5	31.6	24.9	33	85
L2 collection	10/06/21	17.1	28.7	22.0	40	100
	11/06/21	16.3	27.6	22.0	39	98
L3 collection	12/06/21	15.0	30.8	23.4	36	98
	13/06/21	19.3	32.2	24.9	45	100
	14/06/21	18.9	27.3	23.2	47	98
L3/L4 collection	15/06/21	16.2	28.2	22.5	43	97
	16/06/21	15.6	30.1	23.2	45	100
L4/L5 collection	17/06/21	17.4	31.1	24.8	37	96
	18/06/21	18.8	29.8	24.3	50	98
L5 collection	19/06/21	18.7	31.8	25.3	51	100
