# Supplementary material for: Growth Regulation in the Larvae of the Lepidopteran Pieris brassicae: A Field Study
Source: Insects. 2023 Feb 9;14(2):167. doi: 10.3390/insects14020167 (PMC9965483; doi:10.3390/insects14020167)
Supplement: Supplementary file 1 [file insects-14-00167-s001.zip › Baraldi et al Supplementary/Baraldi et al Supplementary Table S3.pdf]

Table S3. Results of one-way ANOVAs for size (*lnCS*) and Procrustes ANOVAs for shape for the extra factor “clutch/plant” at different larval stages (L1-L5) in *Pieris brassicae*. df, degrees of freedom; F, Fisher’s test value; p, p-value.

variable	df	F	p
$size_{L1}$	19	2.30	0.0133
$size_{L2}$	19	3.23	0.0009
$size_{L3}$	19	2.32	0.0124
$size_{L4}$	19	2.19	0.0182
$size_{L5}$	19	2.23	0.0164
$shape_{L1}$	323	1.11	0.1257
$shape_{L2}$	323	1.27	0.0054
$shape_{L3}$	323	1.32	0.0015
$shape_{L4}$	323	1.41	0.0001
$shape_{L5}$	323	1.20	0.0249
